# There must be a way out: The consensual qualitative analysis of best coping practices during the COVID-19 pandemic

**DOI:** 10.3389/fpsyg.2022.917048

**Published:** 2022-09-27

**Authors:** Júlia Halamová, Katarína Greškovičová, Martina Baránková, Bronislava Strnádelová, Katarina Krizova

**Affiliations:** Faculty of Social and Economic Sciences, Institute of Applied Psychology, Comenius University in Bratislava, Bratislava, Slovakia

**Keywords:** compassion, self-compassion, mutual compassion, consensual qualitative research, COVID-19 pandemic, coping

## Abstract

Despite the continuous efforts to understand coping processes, very little is known about the utilization of best coping strategies during the COVID-19 pandemic. In this study, we aimed to analyze the coping strategies of individuals who scored high on an adaptive coping questionnaire in order to understand the most adaptive coping strategies during the COVID-19 pandemic. We used consensual qualitative analysis in a team of four researchers and one auditor. The convenience sample from which we identified the high scorers comprised 1,683 participants (67% women, 32.35% men, and 0.65% did not report their gender) with a mean age of 31.02 years (SD = 11.99) ranging between 18 and 77 years old. Based on their scoring in the COPE Inventory, nine participants were selected from the sample with the highest scores in coping skills in at least two out of its 15 subscales. In-depth repeated interviews with six participants for the main analysis were conducted, and three were added to check the data saturation. The results showed that the most adaptive coping strategies used during the COVID-19 pandemic could be categorized into four main domains: self-compassion, compassion to others, compassion from others, and mutual compassion. The most frequently mentioned and the most elaborated upon by our respondents was the domain of self-compassion. The most interesting finding was the emergence of the fourth type of compassion, labeled *mutual compassion*, which referred to deliberate attempts to take care of oneself and others while suffering together in order to elevate the suffering for both. This kind of compassion might arise in the situations of collective suffering, such as a catastrophe or a pandemic and might have the additional benefit of bringing people closer to each other in difficult times.

## Introduction

The conceptual analysis of stress and coping conducted by Carver et al. (1989) argued that the stress response consisted of three processes: primary cognitive appraisal, during which one evaluates the level of threat, secondary cognitive appraisal, during which one considers their resources and the potential responses to threat, and subsequently, coping, which refers to one’s execution of a specific response that targets the threat demands. In this model, coping is understood as a major part of the stress process, and it is considered to mediate the connection between stress and psychological distress or to moderate the stress–strain relationship (Ogden, 2000).

Previous research on coping strategies can be divided into three areas: examining individual coping strategies, focusing on differing groups of coping strategies, or studying coping profiles which combine different coping strategies (Kavčič et al., 2022). The first one describes distinct basic coping strategies such as 400 strategies gathered by Skinner et al. (2003). The second one refers to the bi-division of strategies in terms of more or less opposite groups of strategies, such as emotion-or problem-focused coping, cognitive or behavioral coping, and direct or indirect coping. Previous studies demonstrated the association between groups of coping strategies and psychological health and well-being (e.g., Park and Adler, 2003). For instance, positive coping styles were found to be related to a lowered risk of anxiety and depression and to higher levels of adjustments in the face of stressful events (e.g., Andrews et al., 1978; Zong et al., 2010; Zhen and Zhou, 2016). The distinction between engagement (aiming at dealing with the stressor, strategies such as problem-solving, planning, acceptance, positive reappraisal) versus disengagement coping (aiming at escaping from the stress, strategies such as denial, withdrawal, avoidance) seems to be the most valuable in terms of physical and mental health (Götmann and Bechtoldt, 2021).

The third area of coping research—combinations of coping strategies-focuses on unique sets of coping strategies referred as coping profiles. People with specific profiles maximize certain coping strategies while minimizing others (Doron et al., 2015). For instance, three profiles evolved from using quantitative measure the COPE or the Brief-COPE Inventories (Carver et al., 1989): a profile with approach-oriented strategies, a profile with avoidance strategies, and a profile with limited strategies (Kavčič et al., 2022). Different profiles might have a differing impact on mental health outcomes. For instance, the profile extensively focused on problem-and emotion-focused coping combined with moderate support seeking and low avoidance coping strategies was found to be associated with lower psychological distress (Eisenbarth, 2012). An identification of distinct coping profiles therefore could be beneficial for both prevention of distress as well as intervention during stressful times (Doron et al., 2015).

### Previous research on pandemic coping in general population

Stress–strain relationships have been investigated by exploring coping strategies in different samples during the COVID-19 pandemic. For example, many previous investigations focus on frontline workers, such as healthcare professionals, nurses, and critical care physicians (e.g., Banerjee et al., 2021) or on vulnerable populations, such as unemployed people (Ogueji et al., 2021a), people with multiple disabilities (Mathias et al., 2020; Galica et al., 2021), or people with mental diagnosis (e.g., Burton et al., 2021). Limited research also focused on general population, for example college students and young adults (Branquinho et al., 2020; August and Dapkewicz, 2021; Vuletić et al., 2021), adults (Lelek-Kratiuk and Szczygieł, 2021; Ogueji et al., 2021b), older adults (e.g., Farhang et al., 2021; Finlay et al., 2021; Greenwood-Hickman et al., 2021; Gonçalves et al., 2022), and families (Salin Voronin et al., 2020).

Several recent studies applied quantitative approach to map coping strategies and their associations with mental health outcomes in general adult populations. Pandey et al. (2022) found that emotional resilience, optimism, and social support were negatively related to negative emotions experienced during the pandemic. Avoidance was found to be effective in minimizing negative emotions, but only for a short time. The mentioned coping strategies were important in returning to normalcy, with an exception of optimism which was found to be important for controlling emotions. The authors suggested there is a difference between escaping strategies and relieving strategies with both leading to different emotional outcomes. Another study highlighted cognitive appraisal and behavioral strategies in adults as essential management tools of psychological distress since they were found to be connected with stable well-being over time (Kim et al., 2022).

Based on the profile approach research, Kavčič et al. (2022) reported three profiles in pandemic coping. An engaged profile encompassed active coping, planning, acceptance, and positive reframing. A disengaged profile referred to low problem-focused coping, social support, acceptance, and positive reframing, and an avoidant profile overused substance use, self-blame, and humor. People with an engaged coping profile scored highest on well-being and lowest on ill-being variables (Kavčič et al., 2022).

Several previous studies utilized qualitative analysis to understand coping during the pandemic. August and Dapkewicz (2021) aimed to understand meaning-focused coping in college students. The most common strategy among college students was found to be *Benefit finding* evidenced by themes such as learning to be grateful, personal growth, and clarity about the future. Additionally, several societal benefits were identified by this study, such as selflessness is widespread, the world has an opportunity to learn what matters, creative solutions and teamwork can help, improvements to the natural environment. In addition to benefit finding, *Negative emotional reactions* strategy manifested in fear, anxiety, and stress, dislike for online learning, and boredom. On the other hand, a theme of hope was found to be part of *Positive emotional reactions*. Comparably, a study by Branquinho et al. (2020) showed that adolescents and young adults were found to cope with the pandemic by having a *regular communication with family and friends via video calls, carrying out pleasurable activities, leading life calmly and positively,* and *having a routine and scheduled times*.

In general adult population, Vuletić et al. (2021) identified several main themes regarding the pandemic: *disruptions in everyday life and functioning, the pandemic as a health crisis, public reaction to the pandemic as a source of tension and frustration, crisis as an opportunity and imagining the post-pandemic future*. Efficient coping strategies that emerged were *focusing on the positive, rational evaluation of the situation and following the preventive measures, proactive approach, and self-organization,* and *seeking support*. On the other hand, inefficient coping strategies were *somatization, passivation,* and *denial and avoidance*. Similarly, Lelek-Kratiuk and Szczygieł (2021) find out that adults generally perceived the pandemic and pandemic-related lockdown as moderate stressors. Stress coping strategies were found to change as a result of increased stress, and in this situation, *avoidance* was presented as the most adaptive reaction. Strategy of acceptance was more present in situation of COVID outbreak in comparison with another stressful situation. In addition, Ogueji et al. (2021b) studied coping strategies of adults and identified 11 themes: *socializing with loved ones (online), engaging in exercise, being occupied with jobs, being occupied with studies, avoiding negative news on COVID-19, consumption of alcohol, healthy eating, engaging in meditation activities, gaming activities, hope, self-care, and self-appreciation*.

Few studies analyzed coping strategies of an older adults. Themes of *belief, faith and hope, family, information/following recommendations* were identified in a sample of elderly people studied by Gonçalves et al. (2022). One of the emerged themes in the study by Farhang et al. (2021) was coping with social isolation/response to difficulties during the pandemics in elderly diagnosed with multiple cognitive impairment. Older participants were found to cope by virtual *connection with friends and family, engaging in community groups and activities, positive attitude, perspective gained from past hardships* (Greenwood-Hickman et al., 2021), but also by *exercise and outdoor* and *daily life activities* (e.g., hobbies, keeping a routine, or reading; Finlay et al., 2021). However, a theme of *nothing* emerged as well, which described participants who reported their inability to cope with stress.

## The aim of the current study

Despite the continuous efforts to understand coping processes and strategies of various populations, very little is known about the utilization of best coping practices during the COVID-19 pandemic. Therefore, in this study, we aim to map and analyze coping strategies of the best copers – the individuals who scored high in adaptive coping on a coping questionnaire (the COPE Inventory; Carver et al., 1989). We posed the following research question:

*How do the individuals, scoring high in coping, cope with the COVID-19 pandemic?*

## Materials and methods

### Measurement instrument

#### The COPE inventory

The COPE Inventory was created by Carver et al. (1989) based on theoretical assumptions about functional coping. The COPE comprises of 60 items and 15 subscales: 1. Acceptance, 2. Active coping, 3. Behavioral disengagement, 4. Denial, 5. Seeking emotional support, 6. Seeking instrumental support, 7. Mental disengagement/Self-distraction, 8. Planning, 9. Positive reinterpretation, 10. Religion, 11. Restraint, 12. Substance use, 13. Suppression of competing activities, 14. Venting, and 15. Humor. The COPE is the most frequently used instrument for measuring of coping behavior (e.g., Voronin et al., 2020) and showed to have good psychometric properties and adequate factor structure in Slovak population (Halamová et al., 2022).

### Research team

Our research team consisted of four postdoctoral researchers in the field of psychology who represented the core coding team and one full professor of psychology who served in the role of the auditor. The professor and two of the core team have been doing research in the field of compassion while the other two members of core team have had experience in coping research.

### Research sample

The convenience sample, gathered online through social media, comprised of 1,683 participants (67% women, 32.35% men, and 0.65% did not report their gender) with mean age of 31.02 years (SD = 11.99). Participants’ ages ranged from 18 to 77 years. All respondents had Slovak nationality and signed an informed consent. After consenting, the participants completed an online questionnaire containing sociodemographic questions and the COPE Inventory (Carver et al., 1989) questions. Since we were interested in the best coping strategies during the pandemic, we randomly chose seven participants from the sample of individuals who had the highest scores on all 15 subscales of the COPE Inventory (Carver et al., 1989). We used the cut-off score of ten points because the maximum score for each the COPE subscale was 14 points. Our selected seven participants had a score higher than 10 points in six, five, four or three subscales of the COPE (Carver et al., 1989). The seventh participant was randomly selected to ensure data saturation after the finalization of consensual qualitative analysis. The high-scoring participants were: a 20-year-old female and a 24-year-old female university students, a 23-year-old female with a secondary education, and a 27-year-old female, a 36-year-old female, a 38-year-old woman and a 46-year-old female with a university education. Since all seven participants were women, we also wanted to check if the categorization were saturated among male participants as well. Therefore, we decided to add two randomly selected male participants who scored highest in the COPE Inventory (more than ten in two subscales). The participants were: a 21-year-old male university student and a 29-year-old male with a university degree. High scoring participants were invited for in-depth interviews. To motivate participants, we offered a 50-euro voucher for the completion of the interview. Data were collected in accordance with the ethical standards of the institutional and/or national research committee and in accordance with the 1964 Helsinki Declaration and its later amendments or comparable ethical standards. The study’s protocol was approved by the Ethical committee of Faculty of Social and Economic Sciences at Comenius University in Bratislava.

### In-depth interviews

We conducted two in-depth interviews with each participant within a two-week timeframe. The interviews with each participant lasted about 180 min. The semi-structured interviews with open-ended questions were focused on the participants’ coping during the COVID-19 pandemic. The interview protocol consisted of the following areas: the participant’s evaluation of their pandemic functioning, recalling of any experienced pleasant situations and the participant’s strategies to savor them, recalling any experienced unpleasant situations during the pandemic and coping strategies that the participant used to deal with them, and the pandemic’s perceived effect on the participant’s lives. The interviews were transcribed verbatim. We used ATLASti.9^1^ for coding and the analysis.

### Data analysis: Consensual qualitative research

First, all researchers reflected in writing on their expectations about the participants’ coping during the pandemic to minimize the biases. Second, the transcribed interviews were analyzed using the Consensual Qualitative Research method (CQR, Hill et al., 1997). This method was primarily developed to investigate inner experiences of complex and rare phenomena that were difficult to capture using the traditional quantitative paradigm. An important feature of this method is that it allows a clear, vivid, and dense description of the phenomenon. This method requires to work in a team. In our case, we had four researchers in the main team and one independent auditor for feedback and control of data analysis. The team approach is used to ensure a diversity of perspectives and thus a greater convergence with the “truth” and a minimization of the researchers’ bias. The CQR approach (Hill et al., 1997) emphasizes the fact that participants are experts in their own experiences and that researchers are the” ones who learn from the participants about the phenomenon. A qualitative approach seemed to be a very appropriate approach for this stage of the study, as it allows the study of inner experience of coping with the pandemic COVID-19 without predetermining the answers of the respondents.

Following the CQR method (adapted from Hill et al., 1997), our research team first identified domains in each of the participants’ narratives. Then, we discussed the domains and reached a consensual solution. The domains were then checked across multiple cases. The research team then constructed subdomains, categories, and subcategories within individual cases and conducted a cross-case analysis. The auditor reviewed the analyzed domains, subdomains, categories, and subcategories of cross-cases, suggested revisions, and discussed with the team. The analysis was done for six cases and then we added one more case to check data stability and then two more male cases to check gender stability of the data. Since added cases showed the analysis to be stable, we finally created a general story of coping with the pandemic within all cases. Following the CQR (Hill et al., 1997), we considered categories which were saturated by all nine participants as general categories, and categories which were mentioned by more than the half (at least by five) participants were called typical. In the final step of our analysis, we received feedback from research participants about the results.

## Results

This study analyzed the coping strategies of participants who scored high on the COPE Inventory in Slovakia during the COVID-19 pandemic. Based on the CQR analysis of nine in-depth repeated interviews, there were 2,477 coded quotations total in 262 unique codes gathered into four domains, 15 subdomains, 36 categories, and 111 subcategories. The most frequent domain was the domain of Self-compassion with 1,896 coded quotations (76.50%), followed by Mutual compassion with 266 coded quotations (10.74%), Compassion to others with 202 coded quotations (8.16%), and Compassion from others with 114 coded quotations (4.60%). We labeled the domains as compassion because they all referred to elevating suffering and distress, even though they differed in the object and the subject of compassion. However, all domains were based on general compassionate competencies derived from both the evolutionary motivational and competencies approach to compassion (Gilbert et al., 2017) and the Strauss et al. (2016) model of compassion incorporating emotions, cognitions, motivations, and behaviors to relieve suffering. Each domain was structured into four subdomains (with an exception of Compassion from others that lacked motivation since we could not assume the motivation of other people to help our participants): thoughts, emotions, motivation, and behavior that participants mentioned in relation to their coping during the pandemic.

### Domain Compassion from others

The domain Compassion from others referred to unsolicited or requested compassion that family, friends, and acquaintances directed towards the participants in various pandemic situations and that the participants recognized as manifestations of compassion. Three subdomains informed this domain: Positive emotions evoked by others; Caring understanding from others; and Caring behaviours by others. In contrast to the other domains, there was no subdomain related to Motivation because the source of compassion was another person, and the participants did not report on other people’s motivation. Please see Table 1 for categorization of the domain with illustrative examples.

**Table 1:** Categorization of the domain Compassion from others.

Compassion from others			
Subdomains	Categories	Subcategories	Examples
Positive emotions evoked by others	Joy evoked by others	Experiencing joy evoked by others interest	*“.. When someone sent me a nice text message, I was so pleased.”*
		Enjoying the presence of one ´s own child	*“My little one is such a joy. It’s like the fact that just looking at that kid and the person’s so happy to have him.’*
	Pride evoked by others		*“(her colleagues sent her flowers because she was sick). It was a surprise, so unexpected, … it was a feeling that I guess what I am doing, I am doing well.”*
	Trust evoked by others		*“Certainly some confidence in the doctor who did the surgery. I mean, I was able to get more information about who he is, what he does. I was able to have conversations with him, get to know him as a person.”*
	Surprise evoked by others		*“But it was definitely such a surprise. So unexpected..”*
	Gratefulness evoked by others		*“.. I have goosebumps as I am saying it but our relationships at work are very close. It’s really like these people care about each other and they thought of me. That was such a wow effect for me.”*
	Satisfaction evoked by others		*“My colleagues are what probably matters most to me in that job. I had a feeling of satisfaction because I put a lot into the job, and now in a way it’s coming back to me.”*
Caring understanding from others	Understanding from others	Understanding from others	*“Because my sister and I have a very good relationship, I knew that if I had a problem with my father, my mother, that I could just talk to her. That no friend will understand me as much as my sister.”*
		Asking others for support	*“.. that I’m going to reach out to people, I’m not afraid to ask for help, I’m just looking for help, I’m just looking for ways to do it, so I can still get something out of it.”*
Caring behaviours by others	Specific care from others	Health care from others	*“I have excellent doctors, a lung doctor and an immunologist. Both called me every day to check on me and to adjust the treatment. I know people at the pharmacy and prepared the medication for me.’*
		Cleaning from others	*“They all came to me to help with cleaning.”*
		Cooking from others	*“My mom has a lot of friends and family members, they would come and bring food, and other things they thought she needed. Those are such, at least for us, pleasant moments.”*
		Purchases from others	*“He had already gone shopping for us, he left it for us at the door and all.”*
		Gifts	*“I was at home after recovering from COVID and they sent me a big bouquet big.”*
	General care from others	Care from others	*“I was able to see who my real friends are, people who texted me to see if I was ok, to check how I felt, whether I needed something.”*
		Initiate a contact with others	*“My other brother started calling occasionally and he would never call before.”*
		Support from others	*“Fortunately, I had documentation from the hospital, and the teacher helped me to complete a request for an extension of the exam period that I needed to submit to the dean. Well, if it wasn’t for her, I probably would not have finished the semester.’*

Two main subcategories emerged from the subdomain Caring understanding from others: Understanding from others which was given freely by other people to our participants and Asking others for support when the participants asked for understanding from other people. In the subdomain Positive emotions evoked by others, six categories about specific positive emotions arose as consequence of other people’s actions towards the participants: Joy, Pride, Trust, Surprise, Gratefulness, and Satisfaction, all evoked by others. The subdomain, Caring behaviours by others, consisted of all statements related to acts of caring from other people towards the participants. The caring behaviors were further divided into two categories: Specific care from others which was related to health care received from others, tidying up, shopping, cooking, gift giving all provided by other people to the participants, and General care from others linked to unspecified caring behaviours from others, such as initiating contact or providing unspecified support to the participants.

### Domain Compassion to others

The second largest domain, Compassion to others, referred to compassion that participants consciously, intentionally, and voluntary directed toward other people in their social network. This domain reflected one-directional manifestation of participants´ compassion toward others which outweighed their own needs and desires and expectations of help in return.

Subdomain Caring thoughts about other people’s suffering was defined as any thoughts that a person had related to their understanding of another’s difficulties. This was represented by one category, Empathy towards others. This category encompassed any empathetic thoughts communicated by the participants when they were imagining the hardships experienced by other people in their lives. Participants often expressed empathetic thoughts when discussing limited or lost opportunities that others experienced due to the pandemic.

Subdomain Caring emotions towards others referred to felt emotions experienced as a result of focusing on difficulties of someone else. Two categories were identified in this subdomain: captured feelings of worry and fear that participants expressed about their loved ones (Worrying about others) and feelings of compassion toward others going through a difficult time (Compassion toward others). The situations that triggered the participants’ compassionate and worrying feelings were usually related to a loved one’s physical and mental health, loved ones’ needs, their home environment, their conflicts with other people, as well as the negative impacts of their loved ones’ being potentially manipulated and believing in conspiracy theories.

We defined subdomain Motivation to care for others as a conscious decision to orient oneself toward another person’s well-being, for example by being there for others, by providing help to others when needed, and by paying attention to others. This subdomain consisted of four categories: Motivation to help other people, Self-sacrifice to maintain relationships, Acknowledgment of others, and Working toward getting along with others. The first category, Motivation to help other people, described the participants’ intentional decision to care for others and help them. Participants explained how they focused on other people’s well-being for example by making sure that their loved ones and friends had a good time and how they were willing to go an extra mile for their family members. The second category, Self-sacrifice to maintain relationships, captured thoughts and behaviours that go against one’s best interest but that one nevertheless engages in so as to keep a relationship with others. For example, the participants described intentionally interpreting tense situations with others as their fault or keeping secrets from others in order to maintain peace in their family. The third category, Acknowledgement of others, referred to participants showing thanks or appreciation towards others, whether by small deeds of caring or by talking about other people in an appreciative and thankful manner. The last category in this subdomain, Working towards getting along with others, captured the deliberate efforts which participants made to maintain good relationships with other people. The efforts fell into two subcategories, Tolerating others and Respecting other people’s individuality. Participants described how they consciously decided to figure out how to get along with others and how to be open-minded and accepting of other’s choices.

The last subdomain in the Compassion to others domain was Caring behaviours towards others. We defined this subdomain as conscious and intentional actions aimed at providing tangible and concrete care to other people or just encouraging them. The participants described specific behaviours that they pursued in order to meet the needs and wants of their loved ones or other people. For example, participants describing grocery shopping for others, purchasing medicine, and communicating with healthcare providers, volunteering, sharing information, providing childcare and pet care, gift-giving, making phone calls on behalf of others, and sharing jokes to cheers others up. All analysis related to Compassion to others is described with specific citations of participants in Table 2.

**Table 2:** Categorization of the domain Compassion to others.

Compassion to others			
Subdomains	Categories	Subcategories	Examples
Caring thoughts about other people’s suffering	Empathy towards others	Expressing empathy toward others	*“There are people who do not have this opportunity and it must be very hard for them.”*
Caring emotions towards others	Worrying about others	Experiencing worry about others	*“I did not get any rest. My husband [sick with COVID] had trouble breathing. At night, through the closed door, I was listening to him sleeping, and I was happy when I heard his snoring. Snoring meant he was breathing normally.”*
	Compassion towards others	Experiencing compassion towards others	*‘I’m not happy that people have more problems, but I’m glad that maybe that’s how I’m going to say it, that people have become more aware of their problems and have started to say they want to solve them.’*
Motivation to care for others	Motivation to help other People	Motivation to care for others’ well-being	*“I wanted everyone to enjoy it, and I did everything that was in my power, so no one felt bored or lonely.”*
		Willingness to help others	*“I was very involved [in helping my sister] because I wanted her to have everything she wished for.”*
	Self-sacrifice to maintain relationships	Self-attribution of responsibility for unpleasant situations	*“I rather think that I did something wrong and [that’s why] the other person reacted the way they did.”*
		Keeping secrets from others	*“It was unpleasant because I got the vaccine, but I could not tell my parents.”*
	Acknowledgment of others	Expressing thanks to others	*“I sent her a message to work expressing my thanks.”*
		Expressing appreciation of others	*“My mom always knew how to go about cooking. How much to make, for how long, all that. When I started cooking, I was wondering how she does it? How is it even possible?”*
	Working towards getting along with others	Tolerating others	*“In our household, we had to learn to get along with each other. There were tense moments but in that year and a half, we had to figure it out and I thought it was possible [despite the tensions].”*
		Respecting other people’s individuality	*“I allowed other people to find their own way.”*
Caring behaviors towards others	Supporting others	Tangibly supporting others	*“I was going grocery shopping for her so she did not need to go to public places.”*
		Encouraging others	*‘.. I encourage others..”*

### Domain Mutual compassion

The domain Mutual compassion contained bilateral and reciprocal compassionate manifestations between the participants and other people. We can define the mutual compassion as win-win situation since both sides gained from these manifestations. The subdomain Mutual understanding referred to mutual exchange of thoughts captured by category Sharing with others. The subdomain Mutual compassionate emotions referred to the participants’ experienced emotional resonance between themselves and other people when they felt close to others or when they reached to others in order to experience closeness. Mutual compassionate emotions were saturated by a specific emotion of mutual happiness, categorized as Shared joy. In the subdomain Mutual compassionate motivation, the participants commented on their striving for living up to their values in their mutual personal and work relationships. Participants described their Pursuit of equal relationship by striving for balance in giving and receiving and also their Belief in good intentions described by the hope that generally, when people act, they do it with the best intentions. The subdomain Mutual compassionate Behavior included a category Seeking out Interactions with people that reflected participants’ efforts to proactively form new relationships and to communicate with others. The subcategory Organizing meetings further reflected the participants’ effort to meet more people and enjoy their presence even in limited pandemic conditions. The second category, Shared activities, captured respondents’ spending time with others by simply being with them or doing activities with them, or engaging in mutual help, for example around household. Participants’ illustrative quotes and the categorization for Mutual compassion can be found in Table 3.

**Table 3:** Categorization of the domain Mutual compassion.

Mutual compassion			
Subdomains	Categories	Subcategories	Examples
Mutual understanding	Sharing with others		*“We called and texted each other a lot, we also sent pictures to each other.”*
Mutual compassionate emotions	Closeness with others	Experiencing of closeness	*“When I come to her and confide in her, we laugh together, we cry together, we are simply on the same wavelength.”*
		Reaching for closeness	*“We had to solve some existential problems recently, that really brought us close to each other.”*
	Shared joy		*“I’m very happy because one of them is pregnant. I am very much part of the pregnancy experience. We are all forward to the baby together and we are going through it together.”*
Mutual compassionate motivation	Being motivated by values	Pursuit of equal relations	*“Since they were worried about me, took interest in me, asked me how I was going and if I needed anything. I wanted to repay the favor. So, I was trying to help them too, I did not know how but at least I was looking for information for them. It was important to me to balance the relationship this way.”*
		Believing in good intentions	“*I believe that we are a close family and we want the best for each other, and that he had a good intention. He did the best he could in the moment.”*
Mutual compassionate behavior	Seeking out interactions with people	Initiate a contact	*“The restaurants opened for the first time. I went with my friends. I initiated us going right away and they agreed.”*
		Find new contacts Organizing a meeting	*“I found a new friend in my class, we were writing to each other during a lecture.”* *“I called two, three friends, and we organized a mini birthday party.”*
	Shared activities	Being with others Doing with others Mutual help	*“We were spending that time very intensively together.”* *“We do things we are able to with a child at home. Or we go outside and explore the nature with our little one.”* *“We spent that time (with my husband) more intensively together. We also took turns taking care of our daughter.”*

### Domain Self-compassion

Self-compassion referred to any manifestations of compassion that was related to participants themselves. All analysis related to Self-compassion is described with specific quotations of participants in Table 4.

**Table 4:** Categorization of the domain self-compassion.

Self-compassion			
Subdomains	Categories	Subcategories	Examples
Self-caring thoughts	Defense mechanisms	Compartmentalization	*“In my mind, I lock it in. I separate it. And if I need to, I’ll let it go.”*
		Denial	*“I pushed it out of my mind.”*
		Suppression	*“If it does not concern me (referring to COVID), I do not need to pay immediate attention to it.”*
		Rationalization	*“I’m trying to rationalize.”*
		Psychological detachment	*“I’m trying to focus on something else.”*
	Acceptance	Acceptance of situations	*“We have gotten used to some things, since it’s basically been going on for a year.”*
		Acceptance of self	*“I’ll admit that this is a weakness of mine. I accept that I have this weakness.”*
		Acceptance of adjustment difficulties	*“It was hard for me to accept. I have not apologized so many times in my life before, did not have to make things right as many times as I did during this period (referring to the pandemic).”*
	Planning	Preparation	*“We were reading medical information about corona we could find.”*
		Planning solutions	*“I turned to a lawyer, looking for help.”*
		Making decisions	*“I’ve made my decision and I want to follow through on it. I want to move on, I do not want to live like this anymore. I’m going to change my life.”*
	Encouraging thoughts	Humor	*“for this corona times, it is already an amazing number of people we can invite. We can have a party with six or seven people, haha!”*
		Empowerment	*“I encourage myself that I can do it.”*
		Boosting by overcoming difficulties	*“For how much we have had to manage in the recent years, this pandemic certainly wasn’t the worst.”*
		Boosting by positive things	*“I realized during the pandemic that it is possible to live very modestly and be quite happy. This was such a radical change for me, that I save more in terms of finances and I am more satisfied with what I have at home, making coffee at home, making breakfast, lunch, dinner at home. If you are at work all day, when you come home you do not want to cook anymore.”*
		Comparing to disadvantaged	*“A lot of people cannot stand it, but I do not mind.”*
		Changing worldview	*“I found that maybe happiness is also easy.”*
Emotions towards self	Mindfulness of emotions	Mindfulness	*“I was aware in that moment, I have a fever, so I am just living in the moment”*
		Mindfulness satisfaction	*“I think I’m a very happy person right now and I think it’s like the pandemic has given me a lot of good.”*
		Not mindful of emotions	*“I am able to turn off my emotions.”*
	Negative emotions towards self	Processing negative emotions	*“I’m going to pay attention to all the bad emotions first.”*
		Crying	*“I am going to let it all out, from the depth of my being.”*
		Helplessness	*“I fall apart. I have no energy, I cannot do anything.”*
		Disappointment	*“.. that’s the disappointment stemming from the system..’*
		Boredom	*“Most of all, I feel bored..”*
		Disgust	*“I really hate all the restrictions.”*
		Dissatisfaction	*“I was terribly dissatisfied for a while and I did not know why..’*
		Loneliness	*“There was a strong feeling of isolation.”*
		Despair	*“.. and then such despair that I have an awful lot of different immunological problems.. that to make it somehow.”*
		Tiredness	*“I’m already overloaded with it and I’m not in control.”*
		Anger	*“I’m angry with those things that are happening and that have happened..”*
		Fear	*“.. dying in hospital is probably one of the worst deaths I could imagine.. that fear..”*
		Sadness	*“.. I was sad about it..”*
	Positive emotions towards self	Savoring	*“I’m going to indulge in different things.. I indulge in food, or I do not know.. I’m going to meet someone good.”*
		Joy	*“I could see how much I have gone through and I’m looking forward to it.”*
		Contentment	*“.. and now I’m going to close the computer and stay in my pyjamas, and I’m going to cook or exercise or read or something. I quite like that it’s like that, I’m fine..’*
		Gratitude	*“Gratitude for the things we have.”*
		Self-compassion	*‘I did not blame myself at all, that was the last thing on my mind.”*
		Fun	*“I get energy from joy and laughter.”*
		Pride	*“Now I have learned how to bake and I had no idea I could do it before.”*
		Self-love	*“.. I just love myself so much.”*
		Hope	*“I also have the hope that maybe something good will happen after all.”*
		Awe	*“With such humility, I accept that it actually worked out somehow..”*
Motivation to self-care	Self-motivation	Self-motivation to mental growth	*“I try to find meaning in things, try to see how things can serve me, but I also try to recognize and accept when a situation is not good for me.”*
		Self-motivation to health and fitness	*“I needed to fight myself to go for a walk, even if the weather was bad, otherwise I would have not gone.”*
	Awareness of one ´s needs	Knowing one ´s needs	*“I became aware of my need for deep connections with other people.”*
		Financial needs	*“I want to focus now on how to get more financial assets, so I do not have to worry if I have enough spending money.”*
		Social needs	*“I need to be around other people, we do not have to be friends, but just being surrounded by others, feeling part of something.”*
		Need for stability and safety	*“she was in the hospital, they did all the examinations for me because my head hurts incredibly.. I wanted to go home explicitly, that I was afraid to stay in the hospital, so I signed the Revers.”*
		Need for activity	*“I needed to move, go for a walk, into the nature, to balance out my tense mental state.”*
		Need for relaxation	*“[After work] I just needed to … observe my surroundings.”*
		Work-life balance needs	*“I was staying at work longer and longer because there was nothing to do outside of work, and I recognized it was not good for me.”*
		Need for self-realization	*“[Pandemic] allowed me to find time for myself… to do what I want to do and not what I have to do. I would like to keep that.”*
		Need for savoring the moment	*“I need to enjoy the sun, the warmth. I know I need this, so I respect this need.”*
		Need for normal pre-pandemic life	*“I miss my morning and evening commute, that was a form of relaxation for me.”*
Self-caring behavior	Self-caring activities	Management of activities	*“What actually helps a lot is to be active. Stay active, do things.”*
		Leisure activities	*“I did not have much time to read before, so now actually, but this year I’m reading my sixth or seventh book.”*
	Religion	Online religious service	*“I can watch a mass online any day.”*
		Community prayer	*“I talked about in my church group, there are six other girls there with me. We prayed for it.”*
		Coping *via* faith	*“I have faith in God, in some greater existence. It actually helps me to be calmer, to take things with more humility.”*
		Relationship with God	*“Dear God, you must save me again. Then he always saves me again.”*
	Active problem solving	Adaptation to the situation	*“Wearing the masks and using the hand sanitizers was the least we could do.”*
		Solving partnership issues	*“The dysfunctional relationship escalated after the summer, while he was staying with me. Then I told him to find another place.”*
		Solving work issues	*“…(positive impact) at a time of online learning, my growth in technology. Such a new experience and the opportunity to work with children and find ways for them to get involved.”*
		Solving health issues	*“I’ve been exercising my back all year because I gave me trouble before. Now I was able to focus on physical therapy and it has gotten a little better.”*
	Setting boundaries	Setting boundaries towards people	*“How else to deal with it, but only in this way. We solved it by meeting only those people who we trusted would protect us with their responsible behavior.”*
		Setting boundaries towards rules	*“I have this principle that I only respect the restrictions as long as they make sense to me. If a rule does not make sense to me, I’m going to break it.”*

#### Self-caring thoughts

Subdomain Self-caring thoughts referred to any kind of rational and mental activity which was aimed to elevate one’s own distress or suffering. In category Defense mechanisms, participants commented on their various ways how to protect themselves from inner conflicts or outer conflicts. The participants used Compartmentalization to keep some thoughts isolated from each other which could together be conflictual. Defense mechanisms also included Denial as the person’s choice to reject reality in order to avoid an unpleasant truth of pandemic or even Suppression of pandemic situation by conscious effort to put disturbing thoughts out of mind, or to control and inhibit these disturbing thoughts and feelings. Participants also spoke directly about the process of giving the logical reasons to justify particular behavior in their minds called Rationalization and about the experience of being mentally away from pandemic, to make a pause in thinking about COVID-related issues and simply to switch off to another mental activity by the mechanism of Psychological detachment in form of mental disengagement or overeating or substance use. Acceptance was another category of Self-caring thoughts represented by tolerance of negative pandemic experiences without trying to change them. Participants accepted either the pandemic situation which is visible in Acceptance of situations or their inner experiences or characteristics which is distinctive for Acceptance of self. The third subcategory of Acceptance was typical by tolerating weaknesses and complications in Acceptance of adjustment difficulties. Planning was also involved in the Self-caring thoughts and included all thoughts related to designing change and better life for the participants. One of the subcategories of Planning was Preparation consisting of all participants´ statements related to information retrieval about the virus and pandemic or even about the current rules, assessing risks, problem analysis etc. Planning solutions was the cognitive active process of setting the specific ways how to manage the situation successfully by for example designing solutions, planning days, events, or the whole life. Utterances referring to the exact and final decision on how to deal with the specific problem were labelled Making decisions. Encouraging thoughts was the most branched category. It involved the use of fun in dealing with the pandemic in subcategory Humor. Subcategory Empowerment was linked to perceiving oneself as strong and resourceful person with options to decide about. Some respondents stated that the pandemic was not the most difficult situation in their lives and therefore they had ability of Boosting by overcome difficulties by remembering already solved and overcome hardship in their previous lives or Boosting by positive things which means that participants reminded themselves positive sides of a difficult situation. Encouraging thoughts were also seen in the of Comparing to disadvantaged by balancing that now it is not as bad as used to be or as someone else is experiencing something worse than me. In the last subcategory of Self-caring thoughts called Changing worldview, there were involved statements related to reassessing past based on the pandemic experiences and transforming their views accordingly.

#### Emotions towards self

The subdomain Emotions towards self encompasses either emotions that were recognized, expressed, or handled with regard to pandemic situations regardless of their positive/negative charge (whether it was connected to pleasant or unpleasant situations). First category which arose in this subdomain was Mindfulness of emotions referring to capacity to notice one’s own emotions, to be receptive to own experience here and now and enjoy being present as well as ability to switch on or off own emotions. The category of Negative emotions towards self consisted of all utterances from participants linked to unpleasant feelings towards self and the qualitative analysis yielded 14 separate subcategories of it: Processing negative emotions referred to ways how to handle negative emotions generally and then there were 13 specific negative feelings such as Crying, Helplessness, Disappointment, Boredom, Disgust, Dissatisfaction, Loneliness, Despair, Tiredness, Anger, Fear, Frustration, and Sadness. The category of Positive emotions towards self consisted of any kind of positive emotion experiencing towards self and composed of 10 separate subcategories: Savoring related to deliberate enjoying pleasant emotions towards self in one’s life, and specific pleasant emotions such as Joy, Contentment, Gratitude, Self-compassion, Fun, Pride, Hope, Awe, and Self-love.

#### Motivation to self-care

The subdomain Motivation to self-care is defined as motivational strategies used by people in order to encourage themselves to engage in self-care activities. Categories Self-motivation and Awareness of one’s needs emerged in this subdomain. In the Self-motivation category, participants described engaging in an internal dialog and self-talk to motivate themselves to act. Motivation strategies were often based on positive encouragement, for example *via* a reward or positive self-talk, but also sometimes on “tough love,” such as when a participant described “fighting themselves” in order to go out for a walk. Participants used self-motivating strategies to work towards mental growth and towards health and fitness. The second category, the Awareness of one’s needs, referred to both the participants’ ability to be mindful to their needs as well as their ability to recognize their needs in various situations and to fulfil these needs. Participants’ responses were also included in this category if they described how they planned on pursuing the fulfilment of their needs. Among the most commonly described needs were Social needs and the Need for activity. Participants also described the Need for life-work balance, and the need for relaxation, financial needs and need for stability and safety. Finally, three unique needs emerged related to the pandemic life’s restrictions: the Need for self-realization, the Need for savoring the moment, and the Need for normal pre-pandemic life.

#### Self-caring behaviour

We defined subdomain Self-caring behavior as conscious, intentional, purposeful and goal directed actions to take care of one’s needs in order to either solve problematic situation that one faces as such or minimize effects of stressful situation on oneself. We identified four categories in this subdomain: Self-caring activities, Religion, Active problem solving, and Setting boundaries. The first category Self-caring activities encompassed all Leisure activities and their management (Management of activities). These activities are deliberately chosen to meet ones needs (such as for physical activity) to diminish the level of stress and positively charged the individuals. They were not carried out to solve the stressful situations. However, they contributed to managing stressful situation as an extra source of happiness, joy, positive emotions, and balanced the negative effect of stressful situations. Participants mentioned a variety of leisure activities such as hobbies, jogging, reading, drawing, sewing, gardening, watching online TVs/apps, studying, listening to music and so on. When describing how they organized their leisure time or handled leisure activities they mentioned picking up a new hobby, changing hobbies, keeping the activities they had been doing, but also that they possessed less/more time for these activities.

Religion category referred to behavior related to religion. Religiously active participants followed principles of religion, its practices, and observances, and they were pondered in developing their religious life as a personal asset. Religious active life helped participants to reduce their stress levels and to feel extra support in challenging situations. We identified four subcategories here: Online religious services, Community prayer, Coping *via* faith, and Relationship with God. The first one referred to attending religious services, the second one to praying in the group of religious people. Copying *via* faith describes faith as a personal source of strength and encouragement to overcome obstacles. Lastly, participants explicitly mentioned God and their relationship with him/her as a specific source of copying strategy. They could talk to, turn to, rely on, have faith in, and ask God for help. They also felt grateful to God and experienced their relationship to be deepening.

Third category labelled Active problem solving was defined as any kind of deliberate behavior aiming to handling or solving the stressful situation. We identified three areas of problems participants were dealing with. They were actively engaged in Solving partnership issues, Solving health issues, and Solving work issues. Moreover, in some cases, participants´ solution was Adaptation to the situation because they did not only accept the situation but also changed or modified their behavior to suit the occurring situation.

The last category in Self-caring behavior was named Setting boundaries because it reflected tendencies of participants to define their limits, define themselves against other people or rules and to keep their integrity. Setting boundaries towards people involves conscious sorting out people that participants wanted to meet or be in touch with, declining in meeting people, changing the way they were meeting with others, standing up for themselves. On the other hand, Setting boundaries towards measure/rules referred to judging the rules and participants willingness to follow or consciously break some of them based on their own evaluation of them and decision how much to follow them.

### General coping experience with the COVID-19 pandemic

The general coping experience with the pandemic COVID-19 refers to coping subcategories that were present in interviews of all nine participants. In the time of difficulties during the COVID-19 pandemic, all best copers effectively dealt with the situations by mostly being compassionate towards themselves. This included self-compassionate thoughts related to an acceptance of difficult situations, especially those perceived by participants as out of control. Participants boosted themselves by remembering previous difficulties they overcame (their previous successes), by focusing on positive things (positive reinterpretation), and by comparing to others who they perceived as disadvantaged (especially comparing their new self to their old self). Participants’ self-compassionate thoughts also involved preparing for, and planning for solutions to help them overcome their difficulties and make important decisions. No defense mechanisms were present in the participants. Participants invested more cognitive energy in planning and encouraging thoughts than in de-engagement strategies. Best scorers were self-compassionate in their emotions by practicing mindful satisfaction, savoring positive emotions, and as a consequence, they often felt contentment. On the other hand, they also felt fear during the COVID-19 pandemic. Best copers were motivated to care for themselves by recognizing and fulfilling their social needs and needs for activity. They also wanted to stay healthy and to be fit and many of their activities were planned and goal-oriented. This motivation was reflected in their leisure activities and their management. Best copers invested their energy in the adaptation to the pandemic situation in which they showed proactive approach to situations, such as compliance with pandemic safeguards. Setting boundaries towards other people as a form of social distancing helped them keep their integrity as well as to meet their need for health. On the other hand, boundaries towards other people also help to fulfil social needs because participants were more effective and active in their refined social network.

During the COVID-19 pandemic, best copers coped also by mutual compassion and compassion towards others. Best copers and people in their social network coped mutually by spending time together, doing activities together, and sharing together. During the pandemic, high copers were compassionate towards others by showing empathy to others which means they showed the cognitive ability to be connected to other people’s suffering and to identify and understand other people’s emotions.

### Typical coping experience with the COVID-19 pandemic

The typical coping experience with the COVID-19 pandemic was defined as being present in data for more than half of the participants. The typical experience was described in addition to the general experience which included being compassionate towards self by using self-compassionate thoughts geared towards acceptance of situations, acceptance of self and one’s weaknesses as well as acceptance of the difficulties with adjustment. Participants boosted themselves by remembering previous difficulties (achieving success), overcoming current difficulties by focusing on positive things (positive reinterpretation), and by comparing themselves to disadvantaged ones (especially comparing their new self to old self but also occasionally by comparing self to others and comparing their own situation to other people’s situations). High scorers nurture their empowerment by remembering their confidence in their own skills, their ability to cope, and their sense of humor, sometimes even the self-defeating kind of humor. The participants’ self-compassionate thoughts involved searching for information, making decisions, preparing, and planning solutions for overcoming the difficulties which sometimes required retrograde assessment and sticking with the decision. High copers planned for big and small events, including their day-to-day, in order to better cope with the pandemic. They were courageous enough to reassess their values and even change their worldview, if necessary. High scorers engaged in psychological detachment and suppression. High scorers were self-compassionate in their emotions by savoring positive emotions during various activities, feeling contentment, joy, hope, gratefulness, and processing negative emotions while experiencing fear, anger, despair, disappointment, crying, insecurity, irritation, sadness, tiredness, health worries, and practicing mindful satisfaction in here and now. High scorers were aware of their own needs and motivated to care for themselves in their social needs, needs for activity (especially physical), needs to turn back to normal pre-pandemic life, needs to relax and switch off, work-life balance needs, needs to savor positive moments, and needs for happy endings. They motivated themselves to take care of their health and expressed mental growth and self-realization. High scorers invested their energy into active problem solving by solving health, relational (bilateral) and work (as they worked more) issues as well as adaptation to the pandemic situation. They also managed their activities by changing hobbies, dedicating more time to activities, doing more spontaneous activities, investing into leisure activities (most frequently walking followed by self-education, exercising, and watching movies and series). High scorers did set boundaries towards other people by for example reducing contact or changing the form of meetings and set boundaries toward rules too. Best copers not only were compliant with pandemic safeguards but they also thoughtfully assessed the recommended safeguards and, in some cases, decided to break rules to find a good balance for themselves.

During the pandemic, high scorers also coped by mutual compassion, compassion towards others and by receiving compassion from others. High scorers looked for closeness with others by initiating contacts with their loved ones, by seeking out interactions with others, and by making new acquaintances. During the meetings, the participants recounted mutual sharing of experiences, spending time together, and doing various activities together. High copers were compassionate towards others by showing empathy to others, being tolerant to others, expressing worries about others, being motivated to care for others’ well-being, supported others by practical help, and also by working towards getting along with others when it was difficult during the pandemic. Best copers received compassion from others mainly in form of support, care, and understanding. The received compassion evoked joy in them which means that they did not resist it but rather they accepted it and were able to enjoy it.

## Discussion

In the current study, we aimed to map and structure coping strategies of participants scoring high in the COPE Inventory (Carver et al., 1989) during the COVID-19 pandemic. We used consensual qualitative analysis (Hill et al., 1997) to examine data collected by repeated in-depth interviews with nine of the participants who were the best copers (six participants initially and three added to check the data saturation), all randomly selected. The results showed that the coping strategies of the best copers during COVID-19 pandemic share an overarching theme of compassion. As defined by Strauss et al. (2016), compassion is a construct which includes our emotions, cognitions, motivations, and behaviours to alleviate suffering. We categorized the coping strategies into four main domains. Each domain has to do with compassion, but the object and the subject of compassion differed (from the most frequently mentioned to the least): self-compassion, compassion to others, compassion from others, and mutual compassion. Comparably, other authors divided compassion in a similar way: compassion for others, compassion from others, and self-compassion (e.g., Neff and Pommier, 2013; Beaumont et al., 2016; Gilbert et al., 2017; López et al., 2018).

Previous studies dealing with coping during the pandemic focused mostly on coping in general and tried to identify and understand the most common coping strategies. Studies that focused on adaptive coping reached similar conclusions than our study. For example, in addition to prevalent self-related coping strategies, Ogueji et al. (2021b) found that individuals often used strategies related to other people, such as Socializing with loved ones. This was reflected by our study in which participants described mechanisms of receiving and giving compassion or mutually providing it.

The most frequently mentioned and the most elaborated on was the domain of self-compassion. Tedeschi (2020, p. 1) wrote that „*Negative experiences can spur a greater appreciation for life*” when reflecting on posttraumatic growth in the times of the pandemic. This seems to be true for best copers in our research. Tedeschi and Calhoun (2004) distinguished five domains of posttraumatic growth: greater appreciation of life; improved relating to others; more personal strength; exploration of new possibilities; and intense spiritual growth. All of these domains were reflected in participants’ experiences as they recalled savoring good moments in life, improved relationships with others, empowering of themselves, perceiving strong need for activity and changing their world view. Instead of dwelling on the obvious negatives, best copers strive to turn negatives into positives not only for them, but for others too.

It is a healthy strategy to compare new self to our older versions of self as a way of boosting our self-confidence rather than comparing yourself with others. In comparison, highly self-critical people are likely to compare themselves to ever rising unreachable standards or to privileged others which makes them feel contempt, disgust, and even hatred towards themselves instead of joy, hope, and contentment (Blatt, 2004).

Participants also encourage themselves by focusing on positive things in their life and on positive reinterpretation. They boosted themselves by remembering their previous successes in hardships. These are important strategies that seem to be valuable for well-being. Finding positive meaning is helpful in COVID times and these strategies bring stable well-being over time (Kim et al., 2022). Positive refocusing and reappraisal are negatively associated with depression, anxiety, insomnia, and social dysfunction (Molero Jurado et al., 2021). This was true for our participants who infrequently reported symptoms of depression, anxiety, and insomnia. On the contrary, they used self-caring thoughts, such as acceptance of negative pandemic situations, which was in line with research conducted by Lelek-Kratiuk and Szczygieł (2021) who found that coping strategy of acceptance was more present during COVID outbreak than in other stressful situations. Self-compassionate mental strategies during the pandemic, such as planning, making decisions, and solution seeking were mentioned frequently by our participants. Active cognitive processes involved in deciding on specific ways to manage the difficult pandemic situations showed that our participants had highly developed executive functions as evidenced by their ability to process information well, plan their actions, and execute their plans. Similarly, previous research found that planning was negatively related to depression and social dysfunction (Molero Jurado et al., 2021) and positively related to well-being during pandemic (Götmann and Bechtoldt, 2021). As mentioned, our participants sporadically reported symptoms of depression, rather, they maintained their well-being and stayed active in their communities.

In addition, our best copers were able to identify and communicate their negative emotions well. Schrauf and Sanchez (2004) found out that it is typical for a well-developed emotion vocabulary to be dominant by expression of negative emotions (50%) over positive (30%) and neutral (20%) emotions. Our best copers did not have such a big percentual difference in expressing their positive and negative emotions (43% of positive to 57% of negative emotional expressions), however, the expression of negative emotions was still dominant. We did not categorize expressions of neutral emotions since we had not asked participants to label them on their own. This greater balance between expressions of positive and negative emotions shown in our research might be attributable to deliberate and purposeful focusing on the participants’ good experiences as opposed to bad experiences. As Schrauf and Sanchez (2004) explained, people generally process positive emotions schematically since they do not pay much of attention to them because they just signal that everything is okay. Negative emotions are, however, processed differently because they indicate that something is not okay and thus, they need more attention and more thinking which results in higher verbal expressivity. Since these results were found to be cross-culturally invariant, we propose they might be attributed to the differences in coping between general population and the best copers.

Other assets of high copers might be their ability to work with their emotions: be mindful, recognize and describe their emotions, process their emotions, and use them as a source of information for improving well-being. If the emotions were pleasant, our participants described savoring them. If they were unpleasant, our participants dealt with them. The participants allowed themselves to feel their emotions whether they were positive or negative and, thus, they were more compassionate towards themselves. Similarly, Greenberg (2011) suggested that people who were aware of all their emotions were more psychologically healthy. These findings are also in line with Gilbert et al. (2012) who suggested that resistance to affiliative and positive emotions is linked to self-criticism and all sorts of psychopathology. Other studies (e.g., Starr et al., 2020; Nook, 2021) also link high emotion differentiation with well-being and low emotion differentiation with psychopathology. Healthy people seemed to be the ones who can feel and be aware and process all sorts of emotions which makes them better prepared to deal with the stressful situations in general (e.g., Kashdan and Rottenberg, 2010). Emotionally resilient and optimistic people had less negative emotions relating to pandemic (Pandey et al., 2022).

Self-compassion and compassion to others is associated to positive affect (Gilbert et al., 2012) while fear of self-compassion and compassion from others are related to self-criticism, depression, anxiety, and stress. This has also reflected in our study. Best copers in our study were also highly compassionate to others as they take care of others and enjoy it and also highly self-compassionate people as they were able to enjoy care and interest from others.

One of the most interesting findings is the existence of a fourth type of compassion—Mutual compassion, which refers to deliberately taking care of myself and others simultaneously while suffering together with the aim to elevate the suffering for both of us. This kind of compassion might arise in the situations of collective suffering, such as a catastrophe or a pandemic and might have additional benefit of bringing people closer to each other in difficult times through experiencing intense sense of community (Peck, 1994, 1998). However, this is the first time mutual compassion emerged from the data in comparison to previous two (e.g., Strauss et al., 2016) or three kinds of compassion (e.g., Gilbert et al., 2017) identified in the scientific literature. The results also support previous findings about the needs and strategies of coping in mutual relationships by socializing (Ogueji et al., 2021b), connecting with friends and family, engaging in community groups and activities (Greenwood-Hickman et al., 2021), but also reflect the changes and coping on various levels of social systems—in link with Salin et al. (2020) perspective of relationship level following the mutual compassionate competencies.

In sum, we identified 111 subcategories of coping strategies. These are distinct strategies that people used to cope with the pandemic and possibly with any other stressful situation in their life. Among these, there were adaptive coping strategies that improve level of functioning and are healthy ways of approaching a problem and focus primarily on problem solving. However, participants also used non-adaptive coping strategies, such as overreaction to situations or avoiding stressful situations. Such coping controversy has been previously reported as short-term adaptive coping strategy (Pandey et al., 2022). This is also in line with conceptual framework of coping strategies mentioned by Skinner et al. (2003). Common divisions of coping strategies (such as into problem-focused versus emotion-focused, approach versus avoidance, cognitive versus behavioral, etc.) are not helpful and could be misleading. Groups of coping strategies are not exhaustive and even the definitions of the groups are not clear. Skinner et al. (2003) therefore discourage from using functional and topological distinctions of coping strategies. We also provided evidence for this idea. In this study, best copers are not primarily best copers because they use only problem-focused or adaptive strategies all the time when facing challenges, but they are best copers because they have a variety and a certain number of coping strategies available, and they can literally choose which one is the best for them in any given moment based on their goals and functions of the coping strategy. So, their choice is much easier to be carried out and they do not need to stick with one decision, one coping strategy. They can alter the strategies according to the situation or development of the situation based on their assets and inner and outer resources. Thus, they are more successful in coping with stressful situations.

Analyzing the strategies of high scores allowed us to identify the profiles of most adaptive coping strategies during the pandemic. Our profiles of general and typical best copers are valuable information for researchers as well as practitioners. In addition to applied research that examines best intervention strategies for pandemic coping (Fernández-Ávalos et al., 2021), our study provides a theoretical background to the understanding of what works and can serve as a point of reference for dealing with any potential future pandemics. Our profiles are comparable to engaged coping profiles identified by Kavčič et al. (2022) that are characterized by unique combination of coping strategies-approach-oriented coping with acceptance. The engaged coping profile shows high well-being which is corroborated with results from our research as well.

### Limits

This study has several limitations. First, the findings from our national sample might not be applicable to other countries because different countries followed different sets of safeguards which might have impacted the citizens’ stress levels. Second, the self-reported COPE inventory (Carver et al., 1989) did not include a socially desirable scale that could help to exclude responders consciously or unconsciously willing to be perceived in a better way, therefore, we were reliant on the participants’ subjective reports of their coping strategies. Third, our sample consisted of volunteers. It has been shown that volunteer participants could differ considerably from general population. Volunteers in research tend to be more interested in the research topic and are more sociable (Rosenthal and Rosnow, 1975). Both of these characteristics could alter our results. Moreover, our initial sample of best copers were all women (even though they were randomly selected). Female participants seem to be more common in psychology research (e.g., Davis et al., 2012; Friesen and Williams, 2016). We included two men in an attempt to balance our sample. Further limitations are also related to our sample, specifically to its size, which was relatively modest, and its socio-demographic indicators. In addition to most of the participants being female, the participants were also in 20–46 age range and had a medium-high education level which might have serve as an advantage in dealing with the pandemic compared to individuals with lower socio-economic status. Similarly, other factors might have contributed to our participants’ best coping strategies that were not considered in this study, such as personality traits (Rettew et al., 2021) and religion (DeRossett et al., 2021).

Finally, the last two limitations relate to the researchers who conducted the interviews. All researchers were females in young or middle adulthood. Based on their traditionally dominant gender norms, however, female interviewers might be more likely to help male participants with articulating and expressing their emotions (Lefkowich, 2019). We also did not do face-to-face interviews because of COVID restrictions, and it might be harder to build rapport in communication *via* phone and video-conferencing, which might have led to our participants being less open during our interviews.

### Future research

Research focused on understanding coping strategies of high copers during other types of stressful events could expand the list of best coping practices. Similarly, having cross-cultural samples to determine whether there are any general or variant coping strategies related to different cultural contexts would be useful. It would also be important to compare the best copers with the worst copers to potentially inform prevention and intervention programs.

### Implication for practice

According to the findings of this study, interventions aimed at cultivating emotional intelligence and resilience, mindfulness, compassion, and flexibility in various coping strategies might be very beneficial for mental and physical health of people during a pandemic or a similar global catastrophe. In developing such interventions, researchers might be inspired by the ways of how the best copers dealt with the pandemic COVID-19 in our study.

## Conclusion

People with high coping skills can thrive even in difficult situations, such as global pandemic. They are able to process their negative emotions, savor positive emotions, motivate themselves to engage in self-care, focus their thoughts to empower themselves, actively solve problems that are solvable and accept the problems that are out of control, and compassionately take care of not only their own needs but also of mutual needs and needs of other peoples. On the top of that, high copers are also able to enjoy compassion from others.

## Data availability statement

The original contributions presented in the study are included in the article, further inquiries can be directed to the corresponding author.

## Ethics statement

The studies involving human participants were reviewed and approved by the Ethical Committee of Faculty of Social and Economic Sciences of Comenius University in Bratislava. The patients/participants provided their written informed consent to participate in this study.

## Author contributions

KK, KG, BS, and MB: writing – original draft, data curation, data analysis, and writing – review and editing. JH: conceptualization, writing – original draft, data analysis, auditor, writing – review and editing, and funding acquisition. All authors designed the research. All authors contributed to the article and approved the submitted version.

## Funding

This work was supported by the Slovak Research and Development Agency under the contract no. PP-COVID-20-0074. Writing this work was supported by the Vedecká grantová agentúra VEGA under grant 1/0075/19.

## Conflict of interest

The authors declare that the research was conducted in the absence of any commercial or financial relationships that could be construed as a potential conflict of interest.

## Publisher’s note

All claims expressed in this article are solely those of the authors and do not necessarily represent those of their affiliated organizations, or those of the publisher, the editors and the reviewers. Any product that may be evaluated in this article, or claim that may be made by its manufacturer, is not guaranteed or endorsed by the publisher.

## Footnotes

^1^ http://atlasti.com
